# Antioxidant Activity of Ruthenium Cyclopentadienyl Complexes Bearing Succinimidato and Phthalimidato Ligands

**DOI:** 10.3390/molecules27092803

**Published:** 2022-04-28

**Authors:** Michał Juszczak, Magdalena Kluska, Aneta Kosińska, Bogna Rudolf, Katarzyna Woźniak

**Affiliations:** 1 Department of Molecular Genetics, Faculty of Biology and Environmental Protection, University of Lodz, 90-236 Lodz, Poland; michal.juszczak@edu.uni.lodz.pl (M.J.); magdalena.kluska@edu.uni.lodz.pl (M.K.); 2 Department of Organic Chemistry, Faculty of Chemistry, University of Lodz, 91-403 Lodz, Poland; aneta.kosinska@chemia.uni.lodz.pl (A.K.); bogna.rudolf@chemia.uni.lodz.pl (B.R.)

**Keywords:** succinimide, phthalimide, ruthenium metallocarbonyl complexes, DNA oxidative damage, ROS, SOD activity, cell cycle, hydrogen peroxide

## Abstract

In these studies, we investigated the antioxidant activity of three ruthenium cyclopentadienyl complexes bearing different imidato ligands: (η^5^-cyclopentadienyl)Ru(CO)_2_-*N*-methoxysuccinimidato (**1**), (η^5^-cyclopentadienyl)Ru(CO)_2_-*N*-ethoxysuccinimidato (**2**), and (η^5^-cyclopentadienyl)Ru(CO)_2_-*N*-phthalimidato (**3**). We studied the effects of ruthenium complexes **1**–**3** at a low concentration of 50 µM on the viability and the cell cycle of peripheral blood mononuclear cells (PBMCs) and HL-60 leukemic cells exposed to oxidative stress induced by hydrogen peroxide (H_2_O_2_). Moreover, we examined the influence of these complexes on DNA oxidative damage, the level of reactive oxygen species (ROS), and superoxide dismutase (SOD) activity. We have observed that ruthenium complexes **1**–**3** increase the viability of both normal and cancer cells decreased by H_2_O_2_ and also alter the HL-60 cell cycle arrested by H_2_O_2_ in the sub-G1 phase. In addition, we have shown that ruthenium complexes reduce the levels of ROS and oxidative DNA damage in both cell types. They also restore SOD activity reduced by H_2_O_2_. Our results indicate that ruthenium complexes **1**–**3** bearing succinimidato and phthalimidato ligands have antioxidant activity without cytotoxic effect at low concentrations. For this reason, the ruthenium complexes studied by us should be considered interesting molecules with clinical potential that require further detailed research.

## 1. Introduction

In recent years, many different ruthenium complexes exhibiting various chemical and biological properties have been synthesized [1]. Complexes with anticancer potential are of particular interest [2,3,4]. Ruthenium complexes exhibit many features that are desirable among cancer drug candidates. They are less cytotoxic to normal cells compared to cancer cells, in which they can accumulate due to uptake by transferrin receptors. Many of them are active against cells that are metastatic and resistant to conventional chemotherapy. We recently demonstrated that ruthenium complex (η^5^-cyclopentadienyl)Ru(CO)_2_(η^1^-*N*-maleimidato) has anticancer potential [4,5]. This complex was about ten times more cytotoxic to leukemic HL-60 cells compared to normal peripheral blood mononuclear cells (PBMCs). Moreover, complex (η^5^-cyclopentadienyl)Ru(CO)_2_(η^1^-*N*-maleimidato) induced DNA damage and apoptosis in HL-60 cells.

Many ruthenium complexes show antioxidant activity [6,7,8,9,10,11,12]. For example, there are two new ruthenium complexes *cis*-[Ru(NO_2_)(bpy)_2_(5NIM)]PF_6_ and *cis*-[RuCl(bpy)_2_(MTZ)]PF_6_, where bpy = 2,2′-bipyridine, 5NIM = 5-nitroimidazole, and MTZ = metronidazole have antioxidant properties in vitro. It was shown that these complexes reduced lipid peroxidation and decreased intracellular ROS levels with comparable effectiveness to the standard steroidal drug dexamethasone or α-tocopherol [9]. Similarly, radical scavenging studies revealed that ruthenium heterocyclic complexes have high activities toward the neutralization of NO and DPPH^●^ (2,2-diphenyl-1-picrylhydrazyl) radicals [11]. Organoruthenium(II) complexes, synthetized and analyzed by Mohankumar et al. [8], effectively scavenged the DPPH^●^ radicals compared to that of standard control ascorbic acid. Moreover, it was shown that some of these complexes also exhibited an excellent in vivo antioxidant activity as they were able to increase the survival of worms exposed to lethal oxidative and thermal stresses by reducing the intracellular ROS levels. The gene expression analysis revealed that these ruthenium complexes maintained the intracellular redox status and offered stress protection through the transactivation of antioxidant defense machinery genes *gst-4* and *sod-3*, which are directly regulated by SKN-1 and DAF-16 transcription factors, respectively [8].

Studies have also shown that some ruthenium complexes have a protective effect on the cardiovascular system [13]. For example, it was revealed that a ruthenium *p*-cymene complex with quercetin binds to 3-hydroxy-3-methyl-glutaryl-CoA reductase (HMGR) both in silico and in vitro and reduces the activity of this endoplasmic, reticulum-bound enzyme that regulates the early stages of cholesterol biosynthesis [14]. It was also observed that this complex had an activity significantly higher than pure quercetin and comparable to those observed for two model drugs, pravastatin and simvastatin. Other ruthenoflavonoid complexes—[Ru(*p*-cym)(chrysin)Cl] and [Ru(*p*-cym)(thiochrysin)Cl]—showed good anti-aggregating activity in washed platelet samples [15]. The complexes were further demonstrated to interfere with several inter-platelet signaling pathways involved in aggregation, such as the integrin αIIbβ3 inside-out and outside-in signaling paths, the phosphoinositide 3-kinase (PI3K) pathway, and the release of granules. Moreover, Ru[Ru(*p*-cym)(thiochrysin)Cl] has been able to inhibit *in vitro* thrombus formation and it was shown to affect haemostasis in mice [15]. It was also found that a mononuclear ruthenium(II) diimine complex along with dietary intervention possesses cardioprotective effects in high-fat high carbohydrate diet-induced prediabetic rats by ameliorating oxidative stress and antioxidant defense enzymes, reducing MAP, restoring the heart to body weight ratio, attenuating derangement in the lipid profile and reducing cardiac inflammatory markers [16].

**Figure 1:**
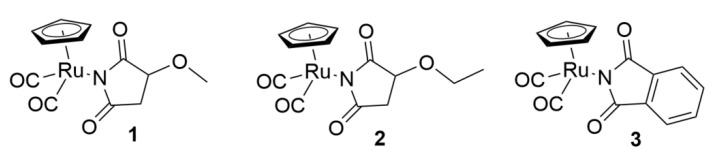
The structures of the ruthenium complexes **1**–**3**.

Recently, we demonstrated that ruthenium complexes (η^5^-cyclopentadienyl)Ru(CO)_2_-*N*-methoxysuccinimidato (**1**), (η^5^-cyclopentadienyl)Ru(CO)_2_-*N*-ethoxysuccinimidato (**2**), and (η^5^-cyclopentadienyl)Ru(CO)_2_-*N*-phthalimidato (**3**) (Figure 1) showed no cytotoxic and genotoxic properties as opposed to the maleimide ligand complex [5]. Moreover, an increase in cell viability was observed after incubation with these complexes. Our comparative studies with free maleimides and succinimides and their derivatives showed that the different biological activity of ruthenium complexes depends mainly on maleimide and succinimide ligands bound to the ruthenium atom [5].

Our previous results prompted us to investigate the antioxidative activity of ruthenium complexes bearing succinimidato and phthalimidato ligands **1**–**3** (Figure 1) in normal and leukemia cells. Many succinimide derivatives synthesized in recent years show anti-free radical properties and are being investigated for their potential use against neurodegenerative disorders such as Alzheimer’s disease, cancer, diabetes mellitus, and worms [17,18,19,20]. Here, we incubated human peripheral blood mononuclear cells (PBMCs) and HL-60 leukemic cells with hydrogen peroxide (H_2_O_2_) to induce oxidative stress. Incubation with H_2_O_2_ was preceded by pre-incubation of cells with ruthenium complexes **1**–**3**. For comparison, we also pre-incubated the cells with ruthenium chloride (RuCl_3_) and succinimide ligand. We investigated the influence of ruthenium complexes **1**–**3** on viability, cell cycle, and DNA oxidative damage. We also determined their effect on the level of reactive oxygen species (ROS) and superoxide dismutase (SOD) activity.

## 2. Material and Methods

### 2.1. Chemicals

Ruthenium complexes **1**–**3** were synthesized as previously described (**1**) [21], (**2**) [22], (**3**) [5]. IMDM medium and fetal bovine serum (FBS) were obtained from Biowest (Cytogen, Zgierz, Poland). Ruthenium chloride (RuCl_3_), 2′,7′-dichlorofluorescein (H_2_DCFDA), Hank’s balanced salt solution (HBSS), dimethyl sulfoxide (DMSO), and hydrogen peroxide (H_2_O_2_) were purchased from Sigma-Aldrich (St. Louis, MO, USA). Succinimide was purchased from Fluka. All other chemicals were of the highest commercial grade available. A stock solution of complexes and succinimide (10 mM) was dissolved in DMSO.

### 2.2. Cell Culture

HL-60 (human promyelocytic leukemia) cell line was obtained from the American Type Culture Collection (ATCC) and cultured in Iscove’s Modified Dulbecco’s Medium (IMDM) with 15% fetal bovine serum, streptomycin/penicillin solution (100 μg/mL and 100 U/mL). HL-60 cells were cultured in flasks at 37 °C in 5% CO_2_ and sub-cultured every 2–3 days to maintain exponential growth.

Peripheral blood mononuclear cells (PBMCs) were isolated from the buffy coats obtained from healthy donors from Central Blood Bank, Lodz. Each donor agreed to donate blood. The first step of isolation of PBMCs was a mix of fresh blood from buffy coats with PBS at ratio 1:1. In the next step, the mixture was centrifuged in a density gradient of Lymphosep (Cytogen, Zgierz, Poland) at 2200 RPM for 20 min with the lowest values of acceleration and deceleration. Then the cells were washed three times by centrifugation with 1% PBS. After isolation cells were suspended in RPMI 1640 medium. The study protocol was approved by the Committee for Research on Human Subjects of the University of Lodz (17/KBBN-UŁ/III/2019).

### 2.3. Cell Viability

The cell viability resazurin assay was performed similarly to the method described by O’Brien et al. [23]. Resazurin salt powder was dissolved in sterile PBS buffer. PBMCs and HL-60 cells were seeded on 6-well plates at a density of 0.5 × 10^6^ cells/mL. Cells were pre-incubated with ruthenium complexes **1**–**3**, succinimide, and RuCl_3_ at a concentration of 50 µM for 24 h at 37 °C in 5% CO_2_. Next, cells were washed with warm PBS. Then, cells were seeded on a 96-well plate in the count of 50,000 for PBMCs and 15,000 for HL-60 cells. Next, cells were incubated with H_2_O_2_ at concentrations of 0.2, 0.4, and 0.6 mM for 4 h at 37 °C in 5% CO_2_. Next, 10 μL of resazurin salt was added to each well, and the plates again were incubated at 37 °C in 5% CO_2_ for 2 h. After that, fluorescence was measured with HT microplate reader Synergy HT (Bio-Tek Instruments, Winooski, VT, USA) using λ_ex_ = 530/25 nm and an λ_em_ = 590/35 nm. The effects of ruthenium complexes, RuCl_3_, and succinimide on cell viability were quantified as the percentage of control fluorescence.

### 2.4. Cell Cycle

HL-60 cells were seeded on 6-well plates at a density of 0.5 × 10^6^ cells/mL. The cells incubated with 100 ng/mL nocodazol (NOC) for 24 h at 37 °C were the positive control. The cells were incubated with ruthenium complexes **1**–**3**, succinimide, and RuCl_3_ at a concentration of 50 µM for 24 h at 37 °C in 5% CO_2_. Next, cells were washed with warm PBS. Then, cells were again seeded on 6-well plates. Next, cells were incubated with H_2_O_2_ at concentrations of 0.1 and 0.2 mM for 24 h at 37 °C in 5% CO_2_. Then, cells were collected and washed twice with PBS. After that, cells were resuspended in PBS and put on ice for 15 min. Then, one volume of −20 °C absolute ethanol was added and the samples were stored at 4 °C. Before the analysis, samples were resuspended in 300 µL of staining solution containing 40 µg/mL PI (propidium iodide) 200 µg/mL RNase A. Samples were incubated for 30 min at 37 °C in the dark until analysis. DNA content was analyzed using an LSRII flow cytometer (Becton Dickinson, San Jose, CA, USA).

### 2.5. Effect of Ruthenium Complexes ***1***–***3*** on DNA Oxidative Damage

We pre-incubated cells with complexes **1**–**3**, succinimide, and RuCl_3_ at a concentration of 50 µM for 24 h in 37 °C in 5% CO_2_, then cells were washed with PBS and incubated with H_2_O_2_ at 0.025 or 0.05 mM for 15 min on ice. Cell viability after incubation with H_2_O_2_ was in the range of 95–100% (data not shown). After incubation with H_2_O_2_ cells were centrifuged, suspended in LMP agarose, and spread onto a microscope slide. The slides were processed as described below in the Section 2.6.

### 2.6. Comet Assay

The comet assay was performed under alkaline conditions according to the procedure of Tokarz et al. [24]. A freshly prepared cell suspension in 0.75% LMP agarose dissolved in PBS was layered onto microscope slides (Superior, Germany), which were pre-coated with 0.5% NMP agarose. Then, the cells were lysed for 1 h at 4 °C in a buffer containing 2.5 M NaCl, 0.1 M EDTA, 10 mM Tris, 1% Triton X-100, pH = 10. After cell lysis, the slides were placed in an electrophoresis unit. DNA was allowed to unwind for 20 min in the solution containing 300 mM NaOH and 1 mM EDTA, pH > 13.

Electrophoretic separation was performed in the solution containing 30 mM NaOH and 1 mM EDTA, pH > 13 at an ambient temperature of 4 °C (the temperature of the running buffer did not exceed 12 °C) for 20 min at an electric field strength of 0.73 V/cm (28 mA). Then, the slides were washed in water, drained, stained with 2 µg/mL DAPI, and covered with coverslips. To prevent additional DNA damage, the procedure described above was conducted under limited light or in the dark.

### 2.7. Comet Analysis

The comets were observed at 200× magnification in an Eclipse fluorescence microscope (Nikon, Tokyo, Japan) attached to a COHU 4910 video camera (Cohu, Inc., San Diego, CA, USA) equipped with a UV-1 A filter block and connected to a personal computer-based image analysis system Lucia-Comet v. 6.0 (Laboratory Imaging, Praha, Czech Republic). Fifty images (comets) were randomly selected from each sample and the mean value of DNA in the comet tail was taken as an index of DNA damage (expressed in percent).

### 2.8. Measurement of Reactive Oxygen Species

To measure reactive oxygen species (ROS), a 2′,7′-dichlorofluorescein diacetate (H_2_DCFDA) probe was used. H_2_DCFDA is a cell membrane-permeable non-fluorescent probe. 2′,7′-dichlorofluorescein diacetate is de-esterified intracellularly and turns into highly fluorescent 2′,7′-dichlorofluorescein upon oxidation. PBMCs and HL-60 cells were seeded at a density of 2.5 × 10^6^ cells/mL and 0.5 × 10^6^ cells/mL, respectively, and were incubated with ruthenium complexes **1**–**3**, succinimide, and RuCl_3_ at a concentration of 50 µM for 24 h at 37 °C in 37 °C in 5% CO_2_. Next, the cells were washed twice with HBSS containing Ca^2+^ and Mg^2+^ and stained with 20 µM H_2_DCFDA (Sigma-Aldrich, St. Louis, MO, USA) for 30 min at 37 °C in darkness. Then, the cells were washed twice with HBSS and incubated with 1 mM and 5 mM H_2_O_2_ at 37 °C in darkness. The intensity of fluorescence was measured after 30 min with λ_ex_  =  495 nm and λ_em_  =  530 nm using a microplate reader Synergy HT (Bio-Tek Instruments, Winooski, VT, USA). The data were analyzed according to the following formula: (T_x_  −  T_0_/T_0_)  ×  100, where T_x_ is the DCF fluorescence measured at the indicated time and T_0_ is the DCF fluorescence measured at the beginning of the analysis.

### 2.9. Measurement of SOD Activity

PBMCs and HL-60 cells were seeded at density 3 × 10^6^ cells/mL and 1 × 10^6^ cells/mL, respectively, in a 75 cm^2^ cell culture flask. The cells were incubated with ruthenium complexes **1**–**3**, succinimide, and RuCl_3_ at a concentration of 50 µM for 24 h at 37 °C in 5% CO_2_. Next, HL-60 cells were washed with PBS and incubated with 0.1 mM H_2_O_2_ for 15 min on ice. In the case of PBMCs, we used 0.25 mM H_2_O_2_ for 15 min at 37 °C. Next, cells were sonicated under the ice in 0.5 mL of PBS for 30 s using the 4710 Series Ultrasonic Homogenizer (Cole-Parmer Instrument Co., Chicago, IL, USA) to obtain cell lysates. SOD activity was measured using the SOD Assay Kit-WST (Dojindo, Kumamoto, Japan) by following the manufacturer’s instructions. Cells lysates served as the sample solution. The mixture was placed into each well with 200 µL of WST working solution. Twenty microliters of enzyme working solution were added into each well and mixed thoroughly. The plate was incubated at 37 °C for 20 min. The absorbance was read at 450 nm using a microplate reader Synergy HT (Bio-Tek Instruments, Winooski, VT, USA).

### 2.10. Statistical Analysis

Data of cell viability, ROS measurement, cell cycle, and SOD activity are presented as the mean values ± standard deviation (SD) of at least three replicates. Values in the comet test are expressed as mean values + standard error of the mean (SEM) of three experiments; data from three experiments were collected and statistical parameters were calculated. Statistical analysis was performed using the Mann–Whitney test (samples with distributions departing from normality) and the Student *t*-test (normal distribution of the sample). Differences were considered statistically significant when the *p*-value was <0.05.

## 3. Results

### 3.1. Cell Viability

All experiments were performed with ruthenium complexes **1**–**3** at a concentration of 50 µM. We chose this concentration based on our previous studies in which we showed an increase in cell viability for both PBMCs and HL-60 after 24 h incubation with ruthenium complexes at 50 µM [5].

**Figure 2:**
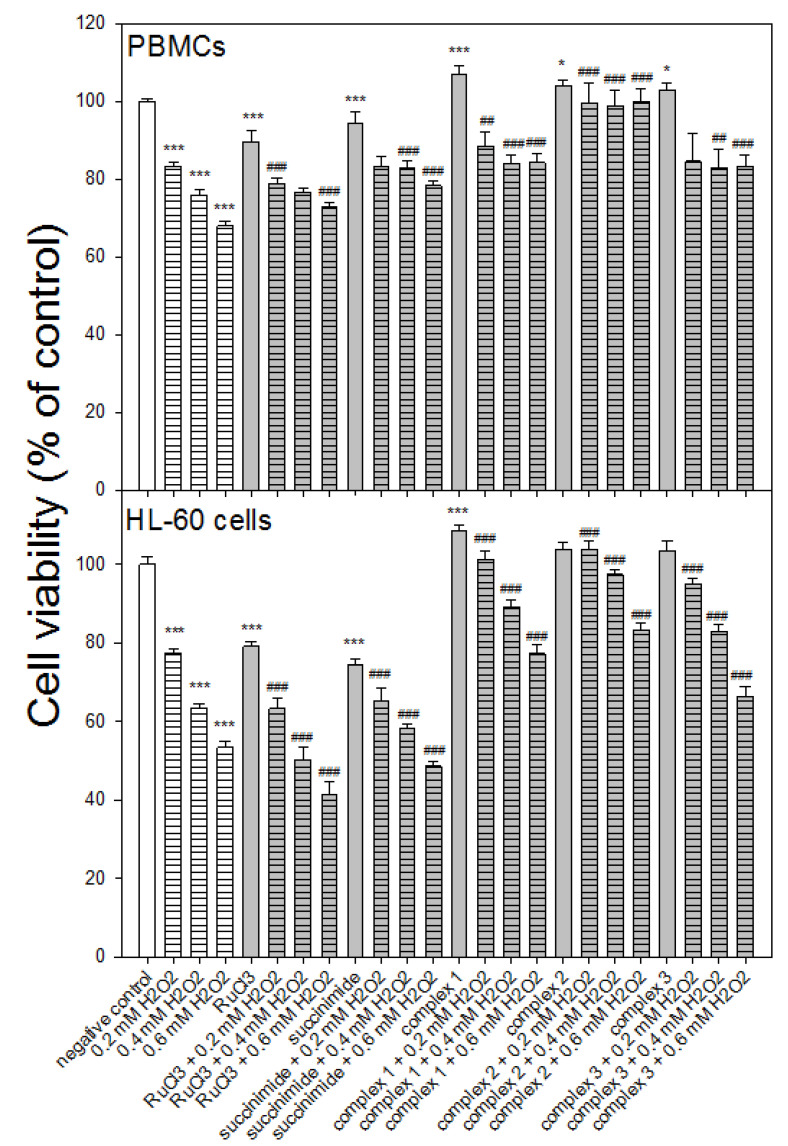
Effect of ruthenium complexes **1**–**3** at 50 µM on the viability of PBMCs and HL-60 cells incubated with H_2_O_2_. The viability for individual samples was calculated relative to negative control (untreated cells) ± SD. Cell viability in the control was taken as 100%, *n* = 6, * *p* < 0.05, *** *p* < 0.001 vs. negative control, ^##^ *p* < 0.01, ^###^ *p* < 0.001 vs. H_2_O_2_.

After pre-incubation, the cells were incubated with H_2_O_2_ for 4 h and the viability was determined with a resazurin reduction assay. We observed a significant increase (*p* < 0.001) in the viability of normal and cancer cells pre-incubated with all ruthenium complexes compared to the cells that were not pre-incubated (Figure 2). The cells that were pre-incubated with RuCl_3_ under the same conditions showed a further decrease (*p* < 0.001) in viability following incubation with H_2_O_2_, except for the PBMCs which were incubated with H_2_O_2_ at 0.4 and 0.6 mM. We also observed a decrease (*p* < 0.001) in the viability of the HL-60 cells that were pre-incubated with succinimide. In the case of the normal cells, we detected the opposite effect of pre-incubation with the ligand—an increase in the viability of PBMCs compared to the cells that were only incubated with H_2_O_2_ at 0.4 and 0.6 mM (*p* < 0.001).

### 3.2. Cell Cycle

After incubation of HL-60 cells with 0.1 mM H_2_O_2_, we observed an increase in the number of cells in the sub-G1 (*p* < 0.001), S (*p* < 0.001) and G2/M (*p* < 0.01) phases of the cell cycle and a decrease in the G0/G1 phase (*p* < 0.001) compared to the control cells (Table 1). H_2_O_2_ at a higher concentration causes a further growth of HL-60 cells in the sub-G1 phase (*p* < 0.001) and a significant decrease in the number of cells in the phases G0/G1 (*p* < 0.01) and G2/M (*p* < 0.01).

We showed a decrease in the number of cells in the sub-G1 phase after pre-incubation with ruthenium complexes **1**–**3** compared to the cells incubated only with H_2_O_2_, both at 0.1 and 0.2 mM (*p* < 0.001). A similar decrease in the number of cells was observed in the S phase (*p* < 0.001). In addition, we demonstrated cell arrest in the G2/M phase by pre-incubation of HL-60 cells with complexes **1**–**3** and incubation with 0.2 mM H_2_O_2_.

HL-60 cells that were pre-incubated with RuCl_3_ or succinimide and then incubated with H_2_O_2_ showed similar changes in the course of the cell cycle as the cells pre-incubated with ruthenium complexes **1**–**3** (Table 1).

### 3.3. DNA Oxidative Damage

**Figure 3:**
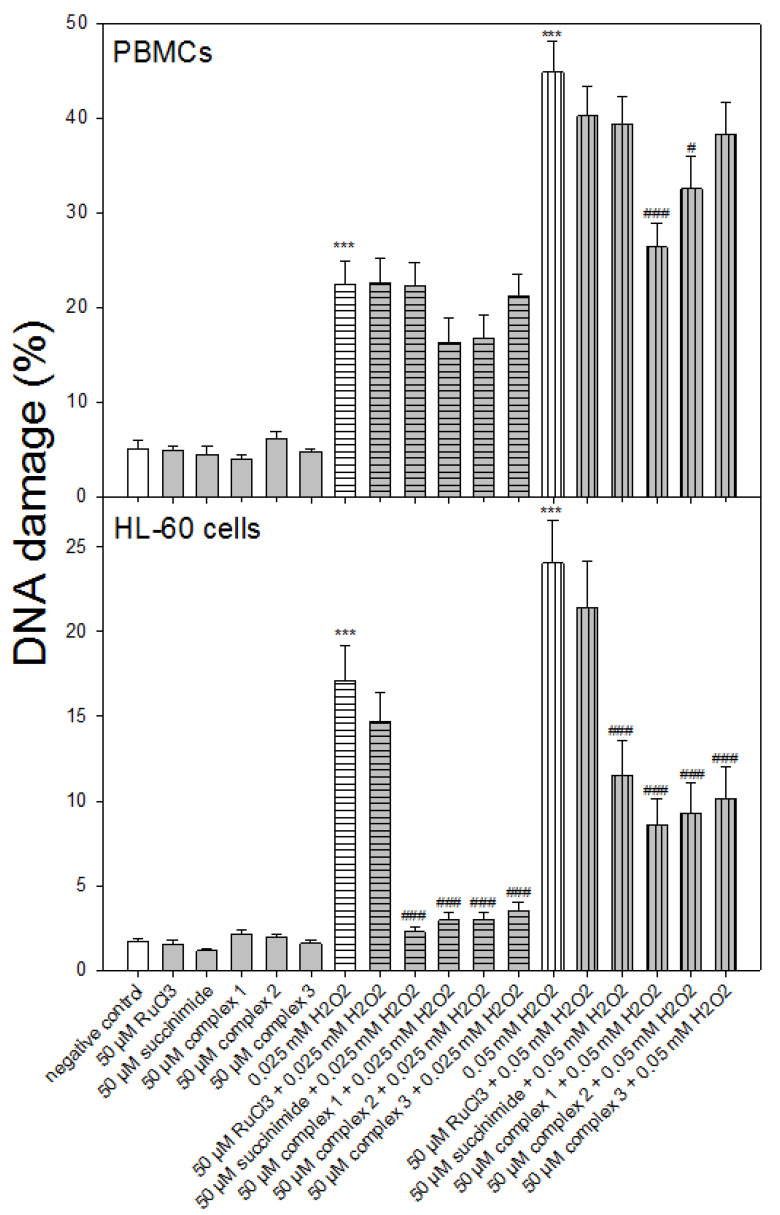
Effect of ruthenium complexes **1**–**3** at 50 μM on H_2_O_2_-induced DNA damage in PBMCs and HL-60. The figures show mean results ± SEM, *n* = 100; *** *p* < 0.001 vs. negative control; # *p* < 0.05, ### *p* < 0.001 vs. H_2_O_2_.

We induced DNA oxidative damage in PBMCs and HL-60 cells using H_2_O_2_ at two concentrations of 0.025 and 0.05 mM. Then we investigated the influence of pre-incubation with ruthenium complexes **1**–**3** on oxidative DNA damage (Figure 3). In the PBMCs we observed a statistically significant decrease in oxidative DNA damage for complex **1** (*p* < 0.001) and complex **2** (*p* < 0.05), but only in the case of incubation with H_2_O_2_ at 0.05 mM (Figure 3). After incubation of the PBMCs with H_2_O_2_ at a lower concentration of 0.025 mM, we did not observe any of the complexes influencing the level of DNA oxidative damage. In the case of the pre-incubation of the PBMCs with both RuCl_3_ and succinimide, we did not detect changes in the level of DNA oxidative damage induced by H_2_O_2_ (*p* > 0.05).

In the experiment with HL-60 cells, we detected a significant decrease in oxidative DNA damage induced by H_2_O_2_ at 0.025 and 0.05 mM for all ruthenium complexes (*p* < 0.001) (Figure 3). We showed a similar effect—reduction of DNA damage in the cells pre-incubated with succinimide and then incubated with H_2_O_2_ at both 0.025 and 0.05 mM concentrations. In the HL-60 cells that were pre-incubated with RuCl_3_, we did not indicate any change in the oxidative DNA damage level (*p* > 0.05).

### 3.4. Reactive Oxygen Species Level

**Figure 4:**
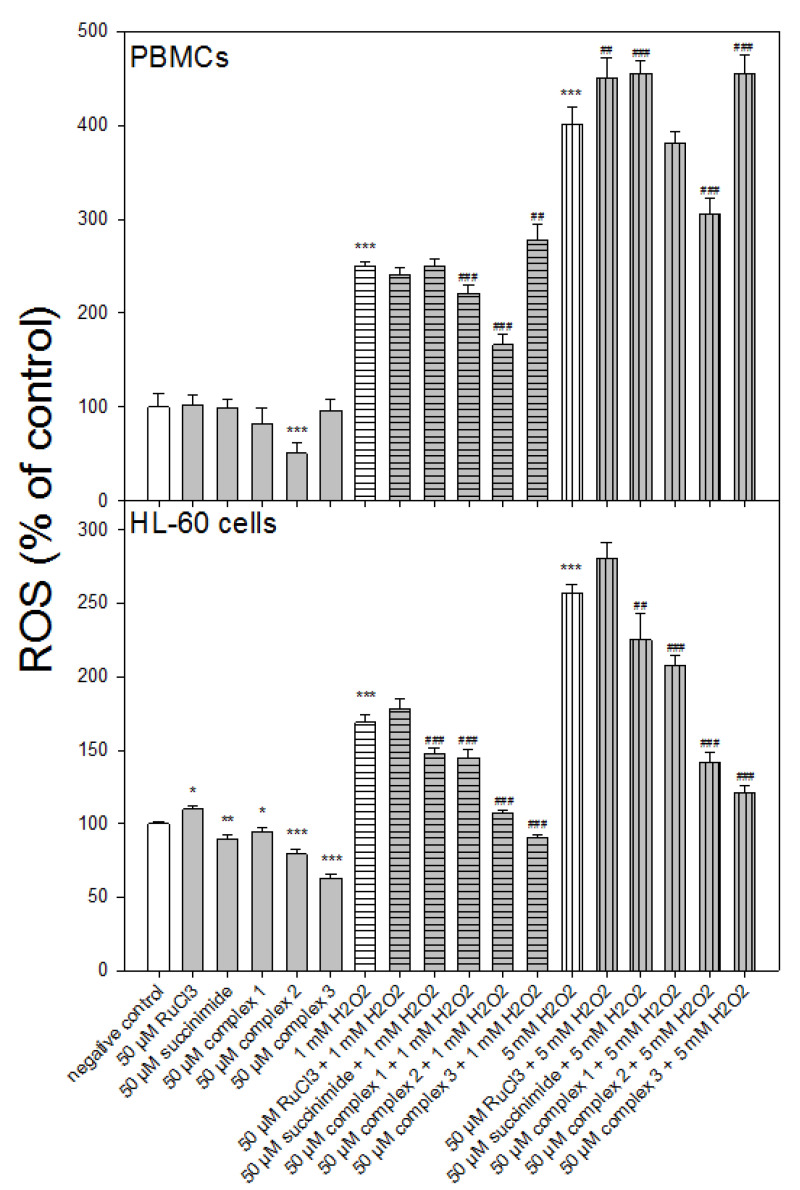
Changes in reactive oxygen species (ROS) level in PBMCs and HL-60 cells pre-incubated with ruthenium complexes **1**–**3** at 50 μM for 24 h at 37 °C and then incubated with 1 mM or 5 mM H_2_O_2_ at 37 °C. Each value represents the mean ± SD, *n* = 6; * *p* < 0.05, ** *p* < 0.01, *** *p* < 0.001 vs. negative control; ## *p* < 0.01, ### *p* < 0.001 vs. H_2_O_2_.

We used an H_2_DCF-DA probe to determine the effect of ruthenium complexes **1**–**3** on reactive oxygen species (ROS) induced by H_2_O_2_ at concentrations of 1 and 5 mM (Figure 4). In the case of PBMCs, we observed a decrease (*p* < 0.001) in the level of ROS for complexes **1** and **2** and 1 mM H_2_O_2_ (Figure 4). In contrast, complex **3** increased the level of H_2_O_2_ induced ROS (*p* < 0.001). A similar effect to the one found in complex **3** was observed in the PBMCs pre-incubated with succinimide and then incubated with 5 mM H_2_O_2_ (*p* < 0.001). Complex **2** has the greatest potential to scavenge ROS in normal cells; it also reduced endogenous ROS levels (*p* < 0.001).

In HL-60 cells, we clearly showed a significant reduction (*p* < 0.001) in ROS levels by all ruthenium complexes (Figure 4). We also observed a similar effect in the HL-60 cells pre-incubated with succinimide and then incubated with H_2_O_2_ at 1 mM (*p* < 0.001) and 5 mM (*p* < 0.01). All ruthenium complexes and succinimide significantly reduced endogenous ROS levels in contrast to RuCl_3_.

### 3.5. SOD Activity

**Figure 5:**
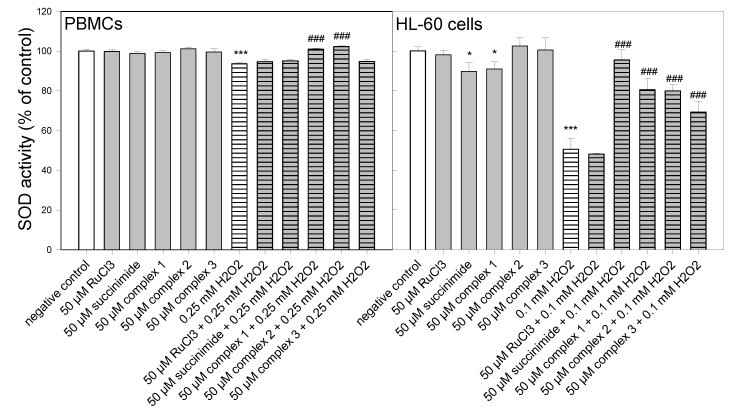
Superoxide dismutase activity in PBMCs and HL-60 cells pre-incubated for 24 h at 37 *°*C with ruthenium complexes **1**–**3** at 50 µM and then incubated with H_2_O_2_ at 0.25 mM in the case of PBMCs and 0.1 mM in the case of HL-60 cells. The figure shows the mean results ± SD, *n* = 3; * *p* < 0.05, and *** *p* < 0.001 vs. negative control; ^###^ *p* < 0.001 vs. H_2_O_2_. Data were normalized to the negative control, which was assigned as 100% of the SOD activity.

In this experiment, we investigated the influence of ruthenium complexes on SOD activity under oxidative stress conditions (Figure 5). First, of all, we observed a decrease in SOD activity (*p* < 0.001) after the incubation of PBMCs (Figure 4) and HL-60 cells (Figure 5) with H_2_O_2_, which corresponds with the results of other research [25,26]. It should be emphasized that in the case of the incubation of HL-60 cells with 0.1 mM H_2_O_2_, we observed a decrease in SOD activity to 50%, whereas in the case of PBMCs the SOD activity decreased only to a level of about 93% after the use of 0.25 mM H_2_O_2_. Further increasing the concentration of H_2_O_2_ to 0.6 mM did not cause any further inhibition of SOD activity in these cells (data not shown).

Our studies clearly showed that all ruthenium complexes increased SOD activity (*p* < 0.001) in HL-60 cells treated with H_2_O_2_ (Figure 5). We observed a similar effect after the pre-incubation of these cells with succinimide (*p* < 0.001). In the case of HL-60 cells pre-incubated with 50 µM RuCl_3_, we did not detect any changes in SOD activity compared to the cells that were not pre-incubated with RuCl_3_. In the case of PBMCs, we observed an increase in SOD activity (*p* < 0.001) after pre-incubation with complexes **1** and **2**. Complex **3** as well as RuCl_3_ and succinimide did not increase SOD activity (*p* > 0.05) (Figure 5).

## 4. Discussion

Here, we investigated the antioxidant activity of three ruthenium complexes with succinimidato and phthalimidato ligands against normal PBMCs and HL-60 leukemic cells. For this purpose, we used 24 h pre-incubation of cells with the complexes and then induced oxidative stress by incubating cells with H_2_O_2_. A correlation and gene ontology pathway analysis identified a rigid association with genes intertwined in cell cycle progression and proliferation after cell incubation with H_2_O_2_ [23]. The ten most substantially correlating genes were validated using qPCR, showing complete congruency with the microarray analysis findings. Western blotting confirmed the correlation of cell cycle-related proteins negatively correlating with H_2_O_2_ IC_25_. It was also shown that the top genes related to ROS production or antioxidant defense were only in modest correlation [27]. The intracellular concentration of H_2_O_2_ is regulated tightly, enabling its use as a cellular signaling molecule while minimizing its potential to cause cellular damage [28]. A high concentration of H_2_O_2_ induces necrosis and a low concentration induces apoptosis. In HL-60 cells H_2_O_2_-induced apoptosis is regulated by decreased BCL-2 [29]. Moreover, it was shown that H_2_O_2_ evoked apoptotic events through the increase of Ca^2+^ in HL-60 cells, inducing caspase -9 and -3 activation, induction of the mitochondrial permeability transition pore (mPTP), and activation of proapoptotic proteins [30].

We observed that H_2_O_2_ at a concentration range between 0.2 and 0.6 mM reduced the viability of normal and cancer cells (Figure 2). In addition, we observed the cell cycle arrest of HL-60 cells in the phase sub-G1 after incubation with H_2_O_2_ at a concentration of 0.1 and 0.2 mM (Table 1). Our results are in line with the results previously reported on the effects of H_2_O_2_ on the HL-60 cell cycle [30]. The arrest of cells in the sub-G1 phase may indicate apoptosis under the influence of H_2_O_2_. This is confirmed by our results on cell viability after incubation with H_2_O_2_. For both PBMCs and HL-60 cells, we observed a significant reduction in their viability. The pre-incubation of both normal and cancer cells with ruthenium complexes **1**–**3** increases their viability (Figure 2) and releases cells from the sub-G1 phase (Table 1).

Hydrogen peroxide, by producing hydroxyl radicals (OH^●^) through the interaction with metal ions near DNA, induces DNA damage such as modified bases, apurinic/apyrimidinic (AP) sites, and single-strand breaks (SSBs). The addition of OH^●^ at position C8 within the guanine ring generates the oxidative product, 8-oxo-7,8-dihydro-2′-deoxyguanosine (8-oxodG). Similarly, the addition of OH^●^ at position C8 of deoxyadenosine generates the oxidative product 8-oxo-7,8-dihydro-2′-deoxyadenosine (8-oxodA). These radicals are capable of further reduction or oxidation forming 2,6-diamino-4-hydroxy-5-formamidopyrimidine (FapyGua) or 8-oxo-7,8-dihydroguanine (8-oxoG), in deoxyguanosine or 4,6-diamino-5-formamidopyrimidine (FapyA) or 7,8-dihydro-8-oxoadenine (8-oxoA) in deoxyadenosine. Another prevalent oxidative product is thymine glycol, produced by the insertion of OH^●^ at position C5 of the thymine rings. Similarly, another oxidation product of cytosine is cytosine glycol, which upon deamination leads to the formation of uracil glycol [31].

Here, we observed a statistically significant decrease in oxidative DNA damage in PBMCs for ruthenium complex **1** (*p* < 0.001) and ruthenium complex **2** (*p* < 0.05) (Figure 3). In the case of HL-60 cells, we noticed a significant decrease in oxidative DNA damage for all ruthenium complexes (*p* < 0.001) (Figure 3). Previously, we detected a protective effect on H_2_O_2_-induced oxidative DNA damage by tricarbonyldichlororuthenium (II) dimer (CORM-2) and the CO-depleted molecule (iCORM-2) [32]. This may indicate that not only the released CO but also the iCORM-2, to which new ligands attach, have antioxidant properties. Here, we also observed that ruthenium complexes reduced the level of ROS induced by H_2_O_2_ in both types of cells (Figure 4). We used the fluorogenic 2′,7′-dichlorodihydrofluorescein-diacetate (H_2_DCFDA) probe to detect ROS. The acetate groups on H_2_DCF allow for diffusion across the plasma membrane, after which both groups are cleaved by intracellular esterases to form H_2_DCF. H_2_DCF is reactive toward many types of oxidants, including nitrogen dioxide (^●^NO_2_), the carbonate radical anion (CO_3_^●–^), the hydroxyl radical (OH^●^), Fe^2+^, Cu^+^, thiyl radicals (e.g., the glutathione radical; GS^●^), and peroxidases (e.g., cytochrome c peroxidase) [33,34]. Importantly, none of the studied ruthenium complexes induced oxidative stress (Figure 4).

Superoxide dismutases (SODs) including MnSOD, Cu/Zn-SOD, and extracellular SOD along with catalase are composed as the first line of defense against ROS. Some studies also showed that oxidative stress downregulated MnSOD in several oxidative stress models induced by H_2_O_2_ and other oxidants. The oxidative stress-induced downregulation of MnSOD activity causes superoxide radical accumulation and superoxide can be converted to OH^●^ in the presence of transition metals (e.g., Fenton reaction) [26]. Research carried out by Gottfredsen et al. [31] showed that *hs*EC-SOD is inhibited and fragmented by H_2_O_2_. The enzyme was inhibited by H_2_O_2_ (37 °C, 1 h) in a dose-dependent manner, with an IC_50_ value of 0.8 mM and complete inhibition at ~2 mM. The mechanism of inhibition was similar to that of bovine Cu,Zn-SOD, including the oxidation of proline (Pro 112) and histidine (His 98, His 163) residues proximal to the active site Cu [35,36].

We detected that all ruthenium complexes increased SOD activity (*p* < 0.001) in HL-60 cells treated with H_2_O_2_ (Figure 5). We observed a similar effect after the pre-incubation of these cells with succinimide (*p* < 0.001). In the case of PBMCs, we observed an increase in SOD activity (*p* < 0.001), decreased by H_2_O_2_, after pre-incubation with complexes **1** and **2** (Figure 5). We showed that ruthenium complexes **1** and **2** reversed the H_2_O_2_-induced downregulation of SOD to normal levels in PBMCs. We assume that ruthenium complexes are responsible for the restoration of SOD activity in the case of PBMCs and its increase in HL-60 cells due to their ROS scavenging capacity. The numerous studies mentioned in the Introduction have shown that various ruthenium complexes synthesized in recent years have the ability to scavenge ROS and RNS [6,7,8,9,10,11,16].

Our results show that ruthenium complexes **1**–**3** bearing succinimidato and phthalimidato ligands at a low concentration of 50 μM protect cells against oxidative stress and do not exhibit cytotoxic activity. Here, we have experimentally confirmed our previous theoretical calculation results on the electronic structure of ruthenium complexes bearing different imidato ligands. We showed that in the case of maleimide, the HOMO-LUMO (highest occupied molecular orbital and lowest unoccupied molecular orbital) energy gap was lower by 1.36 eV when compared with succinimide [5]. Therefore, we suppose that the antioxidant properties of ruthenium complexes, which we studied here, may be due to the high HOMO-LUMO energy gap. Our current comparative studies of ruthenium complexes bearing succimidato ligands with succinimide, and RuCl_3_ indicate that the succinimide ligand, rather than the ruthenium metal center, is responsible for the antioxidant activity of complexes **1**–**3**. We plan to undertake further electrochemical investigations of succinimide and complexes **1**–**3** to shed some light on this problem. Taking into account the fact that oxidative stress is involved in many human diseases, including cancer, antioxidant treatment involving new molecules that exhibit antioxidant properties in biological systems could be of potential interest from a clinical point of view.

## Figures and Tables

**Table 1:** Cell cycle distribution measured by flow cytometry using a staining with propidium iodide in HL-60 cells pre-incubated for 24 h at 37 °C with ruthenium complexes **1**–**3** at 50 µM and then incubated with H_2_O_2_ at 0.1 and 0.2 mM for 24 h at 37 °C. The cells incubated with 100 ng/mL nocodazole (NOC) for 24 h at 37 °C were the positive control.

Treatment	DNA Content %			
	Sub-G1	G0/G1	S	G2/M
negative control	1.19 ± 0.14	35.38 ± 1.64	25.66 ± 0.11	37.21 ± 1.72
positive control (NOC 100 ng/mL)	9.93 ± 2.46 ***↑	22.36 ± 6.91 *↓	21.09 ± 3.53	47.8 ± 4.22 *↑
0.1 mM H_2_O_2_	10.39 ± 0.37 ***↑	11.27 ± 0.88 ***↓	32.73 ± 1.27 ***↑	44.79 ± 0.42 **↑
0.2 mM H_2_O_2_	19.83 ± 0.76 ***↑	27.43 ± 0.62 **↓	23.82 ± 2.54	28.84 ± 2.38 **↓
RuCl_3_	2.02 ± 0.43	35.28 ± 2.03	29.87 ± 0.17	32.5 ± 2.42
RuCl_3_ + 0.1 mM H_2_O_2_	4.73 ± 0.55 ^###^↓	39.25 ± 0.4 ^###^↑	26.12 ± 0.35 ^###^↓	30.02 ± 0.79 ^###^↓
RuCl_3_ + 0.2 mM H_2_O_2_	14.01 ± 0.98 ^##^↓	29.15 ± 1.48	11.6 ± 0.31 ^###^↓	45.37 ± 1.28 ^###^↑
succinimide	2.31 ± 0.33	44.42 ± 3.01 *↑	28.48 ± 2.02	24.17 ± 1.63 ***↓
succinimide + 0.1 mM H_2_O_2_	2.07 ± 0.86 ^###^↓	41.95 ± 3.29 ^###^↑	22.63 ± 3.7 ^###^↓	33.17 ± 1.92 ^###^↓
succinimide + 0.2 mM H_2_O_2_	5.12 ± 0.84 ^###^↓	27.34 ± 2.58	10.76 ± 0.15 ^###^↓	56.63 ± 1.66 ^###^↑
complex **1**	1.76 ± 0.13	48.81 ± 3.48 **↑	27.59 ± 3.97	21.48 ± 1.00 ***↓
complex **1** + 0.1 mM H_2_O_2_	2.22 ± 0.36 ^###^↓	41.71 ± 1.21 ^###^↑	20.29 ± 2.95 ^###^↓	35.32 ± 1.31 ^###^↓
complex **1** + 0.2 mM H_2_O_2_	5.34 ± 1.42 ^###^↓	29.82 ± 3.78	9.72 ± 0.29 ^###^↓	54.96 ± 2.42 ^###^↑
complex **2**	1.69 ± 0.13	49.3 ± 3.58 **↑	26.85 ± 3.54	21.24 ± 0.29 ***↓
complex **2** + 0.1 mM H_2_O_2_	2.5 ± 0.56 ^###^↓	40.92 ± 1.8 ^###^↑	20.59 ± 2.28 ^###^↓	35.59 ± 0.53 ^###^↓
complex **2** + 0.2 mM H_2_O_2_	6.64 ± 1.05 ^###^↓	27.92 ± 0.87	11.3 ± 0.62 ^###^↓	54.24 ± 0.92 ^###^↑
complex **3**	1.76 ± 0.17	51.4 ± 0.78 ***↑	25.21 ± 0.66	21.65 ± 0.38 ***↓
complex **3** + 0.1 mM H_2_O_2_	2.25 ± 0.15 ^###^↓	43.67 ± 1.54 ^###^↑	18.11 ± 1.76 ^###^↓	35.86 ± 1.68 ^###^↓
complex **3** + 0.2 mM H_2_O_2_	4.95 ± 1.23 ^###^↓	29.35 ± 4.14	9.5 ± 1.06 ^###^↓	56.07 ± 2.53 ^###^↑

The table shows mean results ± SD, *n* = 3; * *p* < 0.05, ** *p* < 0.01, *** *p* < 0.001 vs. negative control. ^##^ *p* < 0.01, ^###^ *p* < 0.001 vs. 0.1 or 0.2 mM H_2_O_2_.

## Data Availability

Data on reported results are deposited with the authors.

## Sample Availability

Samples of the compounds (η^5^-cyclopentadienyl)Ru(CO)_2_-*N*-methoxysuccinimidato (**1**), (η^5^-cyclopentadienyl)Ru(CO)_2_-*N*-ethoxysuccinimidato (**2**), and (η^5^-cyclopentadienyl)Ru(CO)_2_-*N*-phthalimidato (**3**) are available from the authors.
